# Late diagnosis among patients with prostate cancer at the Uganda Cancer Institute: a retrospective cohort study

**DOI:** 10.4314/ahs.v24i3.19

**Published:** 2024-09

**Authors:** Nelson Bunani, Angela Nakanwagi Kisakye, Aloysius Ssennyonjo, Fred Nuwaha

**Affiliations:** 1 Makerere University School of Public Health, College of Health Sciences, P.O.Box 7072, Kampala Uganda; 2 African Field Epidemiology Network, Lugogo House Plot 42, Lugogo-Bypass, Kampala Uganda

**Keywords:** Diagnosis, prostate cancer, Uganda, timing, late diagnosis

## Abstract

**Background:**

Late diagnosis of prostate cancer is associated with high mortality, morbidity and low quality of life. We aimed to assess the time of diagnosis among prostate cancer patients in Uganda and investigate the factors associated with early or delayed diagnosis.

**Methods:**

A retrospective cohort analysis of 280 records of patients with histologically confirmed diagnosis of prostate cancer from January 2016 to December 2017. Delayed diagnosis was defined as the diagnosis done at stage III or IV Stage I and II were classified as early. We used modified Poisson regression to assess factors associated with early or delayed diagnosis.

**Results:**

The median from symptom recognition to diagnosis was 12 months (Interquartile Range, IQR: 5-24), with 76% of patients receiving their diagnosis more than 4 months after experiencing symptoms. Notably, 35.7% of patients were diagnosed at stage III, and 46.1% at stage IV. Upon diagnosis, all patients exhibited elevated prostate-specific antgen (PSA) levels with median PSA of 100.2 ng/ml (IQR: 36.02-350) in blood.

**Conclusion:**

Taking a biopsy after 4 months of initial symptoms was partially responsible for the delay in diagnosis. Communities should be educated about prostate cancer symptoms and advised to seek health care early. Health care workers should be sensitized to suspect prostate cancer among patients to allow timely referral.

## Background

Prostate cancer is a significant global public health issue, ranking among the most commonly diagnosed cancers worldwide and accounting for approximately 7.3% of all cancer-related deaths 1. Prostate cancer is the second most diagnosed malignancy and the fifth-leading cause of cancer-related deaths among men globally^2^. Notably, the impact of prostate cancer is particularly pronounced in sub-Saharan Africa, where it constitutes a significant portion of the cancer burden, accounting for approximately 77,300 cases annually, representing 23% of all cancer diagnoses in the region 3.

In Uganda, prostate cancer has been increasing at a rate of 5.2% annually, making it the most rapidly increasing cancer in the country and sub-Saharan Africa^4^-^6^. Despite the increase in incidence, approximately 90% of prostate cancer patients are not aware of the disease, and as a result, they do not take early urinary symptoms seriously; hence, they are diagnosed late with very advanced disease in stage IV, presenting with incurable tumors 4-6. Late diagnosis limits treatment options, increases mortality and leads to low quality of life for patients and their families^7^, ^8^. The factors associated with late diagnosis and, consequently, late treatment have not been well evaluated, especially in low-income countries of sub-Saharan Africa.

Previous studies in Uganda found the median age of prostate cancer diagnosis to be 70 years among Ugandan men, with the majority dying within the first year of diagnosis and only 46.9% living 5 years after diagnosis^9^, ^10^. Early diagnosis of prostate cancer may lead to timely management of the disease, which can lead to better treatment outcomes. Prostate cancer screening is available in Uganda, with the main methods being the PSA blood test and digital rectal examination (DRE)^11^. However, access to screening services varies across regions and healthcare facilities, which limits access 4,12. The cancer diagnosis and treatment infrastructure in Uganda is still developing, with limited resources, a shortage of specialized healthcare professionals, and challenges in accessing treatment, particularly in rural areas, which affects timely screening and diagnosis 13. More efforts are needed to effectively address the growing burden of cancer in Uganda. We conducted this study to assess the time of diagnosis among prostate cancer patients in Uganda and investigate the factors associated with early or delayed diagnosis.

## Methods

### Study setting

We conducted this study at the Uganda Cancer Institute (UCI). The UCI is a comprehensive cancer treatment and research center owned by the government of Uganda. It is part of Mulago National referral hospital complex located along Upper Mulago Hill Road about five kilometers from Kampala central business district. It offers dedicated clinics and specialized departments for all cancers following a multidisciplinary approach involving various healthcare professionals 14. Prostate cancer services at UCI include diagnosis, staging, treatment (such as surgery, radiation therapy, hormonal therapy, chemotherapy, and targeted therapy), and follow-up^11^,^15^. Referrals for prostate cancer patients come from the community, primary care physicians, urologists, and healthcare providers within Uganda and neighboring countries 14, 16. All suspected and confirmed prostate cancer patients in the country are referred to UCI for management.

The guidelines for diagnosis and management of patients with suspected cancer of the prostate cover both symptomatic and asymptomatic cases 15. The guidelines for patients with symptoms state that prostate cancer screening should be considered in any male above 50 years of age and or above 40 years of age if he has a first-degree relative who has had prostate cancer and or is of African ethnicity 17. For asymptomatic men seeking screening, the guidelines recommend counselling to undertake a prostate-specific antigen test and DRE if the prostate-specific antigen is raised 11. Referral to Uganda Cancer Institute is considered after two abnormal prostate-specific antigens at least 6 weeks apart and or abnormal hard prostate on DRE 15. In men in their 40s and 50s, a PSA score exceeding 2.5 ng/ml is considered abnormal, whereas in men in their 60s, a PSA score higher than 4.0 ng/ml is considered abnormal. In such cases, a digital rectal examination (DRE) is recommended as a follow-up procedure. The DRE helps provide additional information about the prostate gland's condition and assists in the evaluation and diagnosis of potential abnormalities or prostate cancer and staging. Prostate cancer staging involves a series of assessments to determine the extent of the cancer within the prostate gland and its potential spread to nearby or distant areas of the body. It includes a clinical assessment, PSA test, imaging studies, biopsy, and classification using the TNM system. The TNM system categorizes cancer based on tumour size and invasion, lymph node involvement, and the presence of metastasis. The resulting stage helps guide treatment decisions.

### Study design

This was a retrospective cohort study in which we quantitatively analyzed records of 280 patients with a histologically confirmed diagnosis of prostate cancer at the UCI between January 2016 and December 2017.

### Description of Materials

We abstracted data from records of all newly diagnosed prostate cancer patients registered at the Uganda Cancer Institute from January 2016 to December 2017. These records included patient files, registers, summary sheets, doctors' medical and referral notes. The data was abstracted using a data abstraction tool. All patients reported symptoms at the time of diagnosis, which included frequent micturition, weak flow of urine, urge to urinate frequently at night, blood in the urine, erectile dysfunction and burning sensation during urination. We excluded 15 patient records which did not indicate the date of onset of the initial perceived symptoms and the date when diagnosis was made.

### Study variables

The primary outcome variable was the stage of prostate cancer at the time of diagnosis. Stages 1 and 2 were classified as early, whereas stages 3 and 4 were classified as late. This classification was based on prostate cancer staging by the American Cancer Society 17. Independent variables included time taken to diagnosis, age, family history of prostate cancer, education level, occupation, ethnicity, marital status, religion, co-morbidities and presenting symptoms. The time of diagnosis was obtained from the difference between the recorded estimated time of onset of initial symptoms and the time when a biopsy was done.

### Data management

The data were collected, cleaned and entered into Microsoft Excel 2016. Continuous numerical responses were entered as absolute values, while categorical responses were coded. The data were stored in a confidential manner in a password-protected computer.

### Data analysis

Data were analyzed using STATA version 14. All continuous variables were summarized using medians with interquartile ranges, while categorical data were recorded as proportions with percentages. We used Pearson's Chi-square test to examine the associations between independent variables and the timing of diagnosis among prostate cancer patients. Modified Poisson regression with robust variances was used at bivariable and multivariable analysis to identify factors associated with the timing of diagnosis among prostate cancer patients at the Uganda Cancer Institute. Prevalence ratios (PRs) were used to estimate the strength of association between the outcome and indicator variables, and associations were tested at a 95% confidence interval (CI).

### Ethics approval and consent to participate

We obtained ethical approval to conduct the study from the Makerere University School of Public Health Research and Ethics Committee (MAKSPHREC) and the Uganda Cancer Institute Research and Ethics Committee (UCIREC) (Ethics certificate number REO/AC/002). Patient files were identified using the prostate cancer patient registration numbers, and no personal identifiers were used. We handled all the data we collected and the patient files with confidentiality.

## Results

### Time of diagnosis among prostate cancer patients in Uganda

The median time from symptom recognition to diagnosis for the prostate cancer patients at Uganda Cancer Institute between January 2016 and December 2017 was 12 (IQR5-24) months. Out of the 280 patients seen at the UCI between January 2016 to December 2017, only 24 % were diagnosed within the first four months of perception of the symptoms. Their median time to admission at Uganda Cancer Institute from the initiation of symptoms was 14 (IQR 6-24) months. Furthermore, more than three-quarters (81.8%) of the patients were diagnosed late, of which 35.7% were in stage III and 46.1% were in stage IV (Table 1).

**Table 1:** Time of diagnosis and histo-pathological findings of the prostate cancer patients diagnosed at Uganda Cancer Institute, January 2016 and December 2017

Diagnosis & histo-pathological findings	Frequency	%	Median	IQR
**Time to diagnosis from first perceived symptoms**			12	5-24
1-4 months	68	24.0		
5+months	212	75.7		
**Cancer stage**				
1	41	14.6		
2	10	3.6		
3	100	35.7		
4	129	46.1		
**Gleason score**				
≤ 6	41	14.6		
7	10	3.6		
8	98	35.0		
9	76	27.1		
10	55	19.6		

### Characteristics of prostate cancer patients diagnosed between January 2016 and December 2017 at the UCI

Overall, prostate cancer patients presented with very high prostate specific antigen levels with a median PSA of 100.2 (IQR 36.0-530) nanograms of PSA per milliliter (ng/mL) of blood. Their median age at the time of diagnosis was 70 (IQR 66-74.5) years. Eighty three percent of the patients were married and at least 85% had other comorbidities which included hypertension, diabetes type 2, Gastroesophageal reflux disease (GERD), urinary tract infections, and urinary retention. Catholics constituted 129 (47.3%) of the patients and most of the patients 197(78.5%) had no family history of prostate cancer. Less than half of the patients 113(40.4%) were Baganda and they were mainly peasant farmers. Only 24% of the diagnosed patients had a digital rectal exam done as a way of testing for prostate cancer. These included frequent micturition, weak flow of urine, urge to urinate frequently at night, blood in the urine, erectile dysfunction and burning sensation during urination. At the end of the 2 years 63.2% of the patients had been lost to follow up, 12.1% were still in care and 24.7% had died. More than three quarters of the patients 273(86.2%) had metastases, which were mainly in the bones, lymph nodes, rectum, liver, and the bladder (Table 2).

**Table 2:** Characteristics of prostate cancer patients diagnosed between January 2016 and December 2017 at the UCI

Factor	Number (%)	Median	IQR
Baseline PSA level		100.2	36.02-350
**Age**			
45-64	68(24.3)		
65-74	140(50.0)		
75-95	72(25.7)		
**Marital status**			
Not married	49(17.5)		
Married	231(82.5)		
**Comorbidities ±**			
No comorbidities	42(15.2)		
Comorbidities	235(84.8)		
**Family history**			
No	197(78.5)		
Yes	54(21.5)		
Religion*			
Catholic	129(47.3)		
Protestant	122(44.7)		
Muslim	22(8.0)		
**Ethnicity**			
Baganda	113(40.4)		
Banyankore	29(10.4)		
Mukiga	36(12.9)		
Gishu	39(13.9)		
Others	63(22.5)		
**Region**			
Western	100(35.7)		
Central	121(43.2)		
Eastern	25(8.9)		
Northern	34(2.1)		
**Occupation**			
Peasant farmer	156(55.7)		
Civil servants	66(23.6)		
Others	58(20.7)		
**Symptoms**			
Lower urinary symptoms	176(64.5)		
Bone pain	30(11.0)		
Others	67(24.5)		
**Digital Rectal Exam done before referral to UCI**			
Yes	63(24.4)		
No	195(75.6)		
**Patient current status**			
Alive and in care	34(12.1)		
Dead	69(24.6)		
Lost to follow up	177(63.2)		
Bone metastasis*			
No	38(13.8)		
Yes	273(86.2)		

* *Variables that had missing data*. **± Comorbidities**

Most of the patients had low urinary symptoms. Digital rectal was done in some health facilities that provide prostate cancer screening and at the Uganda Cancer Institute. Other symptoms included blood in urine, erectile dysfunction, general body weakness, fever, malaise and loss of weight. Areas of metastasis included bones, lymph nodes, rectum, liver, and the bladder.

### Bivariable and Multivariable analysis of the factors associated with early or delayed diagnosis among patients with prostate cancer at UCI

At bivariable analysis, only the time taken to request biopsy from the time first symptoms appeared significantly associated with early diagnosis with a P-value of 0.012. (Unadjusted PR 2.18, 95% CI 1.34-3.54, P=0.012).

At the multivariable level, biopsies made/taken within 4 months of recognizing symptoms were associated with early or late diagnosis among patients with prostate cancer. After controlling for age, comorbidities, religion and presenting symptoms, the chance of being diagnosed early was 2.04 times among patients who had a prostate biopsy within 4 months from the time they felt the initial symptoms compared to those who had a biopsy after four months from the time they felt the first symptoms (adjusted PR 2.40, 95% CI 1.46-3.96, P= 0.001) (Table 3).

**Table 3:** Bivariable and Multivariable analysis of the factors associated with *early or delayed* diagnosis among patients with prostate cancer at UCI

Factor	Number and % of patients diagnosed early	Number and % of patients diagnosed late	Unadjusted PR (95% CI)	Adjusted PR (95%
**Time taken to seek care from first perceived symptoms**				
1-4 months	21(30.9)	47(69.1)	1	**1**
5+months	30(14.2)	182(85.9)	2.18(1.34-3.54) *	**2.40 (1.46-3.96)**
**Age**				
45-64	15(22.1)	53(77.9)	1	1
65-74	26(18.6)	114(81.4)	1.04(0.90-1.21) ^ns^	1.05(0.91-1.21)
75-95	10(13.9)	62(86.1)	1.10(0.94-1.29) ^ns^	1.11(0.95-1.31)
**Marital status**				
Not married	10(20.4)	39(79.6)	1	
Married	41(17.8)	190(82.3)	1.03(0.89-1.21) ^ns^	
**Comorbidities**				
No comorbidities	11(26.2)	31(73.8)	1	
Comorbidities	39(16.6)	196(83.4)	1.13(0.93-1.37) ^ns^	1
**Family history**				1.19(0.98-1.44)
No	32(16.2)	165(83.8)	1	
Yes	12(22.2)	42(77.8)	0.93(0.79-1.08) ^ns^	
**Religion**				
Catholic	18(14.0)	111(86.1)	1	
Protestant	25(20.5)	97(79.5)	0.92(0.82-1.04) ^ns^	
Muslim	7(31.8)	15(68.2)	0.79(0.59-1.06) ^ns^	
**Ethnicity**				
Baganda	24(21.2)	89(78.8)	1	
Banyankore	4(13.8)	25(86.2)	1.09(0.92-1.30) ^ns^	
Bagishu	9(25)	27(75)	0.95(0.77-1.18) ^ns^	
Bakiga	4(10.3)	35(89.7)	1.14(0.99-1.31) ^ns^	
Others	10(15.9)	53(84.1)	1.07(0.92-1.23) ^ns^	
**Region**				
Western	17(17.0)	83(83.3)	1	
Central	24(19.8)	97(80.2)	0.97(0.85-1.10) ^ns^	
Eastern	3(12.0)	22(88)	1.06(0.89-1.26) ^ns^	
Northern	7(20.6)	27(79.4)	0.96(0.79-1.16) ^ns^	
**Occupation**				
Peasant farmer	26(16.7)	130(83.3)	1	
Civil servants	13(19.7)			
	17.86	53(80.3)	0.96(0.84-1.11) ^ns^	
Others	12(20.7)	46(79.3)	0.95(0.82-1.10) ^ns^	
Digital Rectal Exam done *				
Yes	8(12.7)	55(87.3)	0.92(0.82-1.04) ^ns^	
No	38(19.5)	157(80.5)	0.87(0.79-0.96) ^ns^	
**Presenting symptoms**				
Lower urinary symptoms	29(16.5)	147(83.5)	1	1
Bone pain	4(13.3)	26(86.7))	1.04(0.89-1.21) ^ns^	1.06(0.91-1.24)
Others	17(25.4)	50(74.6)	0.89(0.77-1.04) ^ns^	0.89(0.77-1.03)

* **P< 0.05**

ns: **not statistically significant; PR-Prevalence Ratio**

## Discussion

We found that the median time from symptom recognition to diagnosis for prostate cancer patients was 12 months. This time was slightly higher than what was reported in Burkina Faso, where prostate cancer patients spent an average of 11 months to get diagnosed 18. Most of the patients were diagnosed with very high levels of prostate-specific antigen and metastatic cancer. The raised PSA levels may be linked to the advanced age 19. Furthermore, patients who had biopsies within 4 months of initiation of symptoms were two times more likely to be diagnosed early compared to patients who had biopsies after 4 months. This is comparable to studies conducted in Florida, USA and Toronto, Canada, which found that men diagnosed with early-stage prostate cancer had a shorter wait time to diagnosis^20^-^22^. Our study findings further align with the work of Garufi 23, who observed that the utility of multiparametric magnetic resonance imaging targeted biopsy investigations may be diminished when performed significantly later than the initial indication for biopsy, based on clinical parameters such as digital rectal DRE and PSA levels.

Late diagnosis could have been due to patients' delay in seeking health care for the symptoms or due to health systems delays in requesting and performing the necessary investigations, including prostate biopsies. The possible causes of patient delay include lack of knowledge regarding the importance of symptoms, trivialization of symptoms, financial and economic barriers, poor health-seeking behavior for men, lack of trust in health systems and seeking care from non-professionals such as traditional healers, spiritual healers and Chinese herbalists 23, 24. On the other hand, health system delays may be attributable to an inadequate number of healthcare workers able to attend to patients, lack of knowledge and skills to assess patients, limited diagnostic equipment as well as lack of supplies, delayed referrals, and health workers not considering cancer of the prostate in their differential diagnosis. Related studies in low and middle-income countries also found an association between delays and late diagnosis among cancer patients 23, 25. Such late diagnosis results in poor prognosis and death^26^.

Related studies in Uganda and other developing countries have found other possible causes of patient and health system delays to include patients' non-adherence to the recommended investigations, referral complexity as cancer investigations are done from different locations and lack of large-scale population-based prostate cancer screening 4, 12, 27. Indeed, in our study, all patients were assessed for prostate cancer after developing symptoms. This explains why most of the patients presented with late diagnosis. Even among patients in whom biopsy was done within 4 months of symptoms, 69% had advanced disease.

We further found that most patients were lost to follow up, while others had succumbed to prostate cancer within two years. The high loss to follow-up may be related to the limited capacity of the Uganda Cancer Institute to provide adequate care and follow-up due to limited staffing levels 28. This implies that the health system in Uganda and the Uganda Cancer Institute need to do more follow-up of patients after confirmatory diagnosis of prostate cancer to improve the prognosis of prostate cancer patients.

The major strength of this study was that it was conducted in a national referral facility which is the only center that provides cancer treatment in Uganda, this helped us to capture data from all the patients across Uganda.

This study neither differentiated between patient and health system delays nor analyzed the reasons for the delays. Future studies are needed to differentiate between patient and health system delays and to elucidate the reasons for these delays. Furthermore, there could have been some confounding by indication of biopsy due to attribution of lower urinary symptoms to an enlarged prostate rather than cancer that could have led to an earlier biopsy and earlier diagnosis especially when digital rectal examinations are rarely done.

## Conclusion and public health implications

Most patients with prostate cancer were diagnosed late. Patients who had a biopsy within 4 months of initiation of symptoms had a higher chance of being diagnosed early. Our data suggest that the community should be educated about the symptoms of prostate cancer and advised to seek health care early. In addition, the healthcare system should be strengthened to detect and diagnose prostate cancer in a timely manner. For instance, healthcare workers should be educated to suspect prostate cancer among their patients so that they are able to refer such patients for appropriate specialized assessment and management. More research is needed to better understand the reasons for the delays and to evaluate interventions aimed at reducing the delay in diagnosis of prostate cancer (e.g.) regarding the feasibility of screening asymptomatic men.
